# Buspirone attenuates cyclophosphamide-induced renal dysfunction in association with alterations in miR-205/EGLN2, Nrf2, and PERK/ATF4/CHOP signaling

**DOI:** 10.3389/fphar.2026.1871321

**Published:** 2026-08-17

**Authors:** Ahmed M. El-Dessouki, Amal Abdullah Alrashidi, Mohammad I. Jumaa, Hagar M. El-Sadek, Asmaa Ramadan, Shaimaa Fathy Mohamed Khalil, Sara Youssif Ibrahim, Nada A. Ashour, Samar H. Rizk, Omneya Galal, Nahed A. Raslan, Ahmed Mohamed Farghly, Amina Ibrahim Badawy, Shaza M. Elhusseiny, Farha M. Shaikh, Wael M. Elsaed, Hend A. Abdallah, Hany M. A. Sonpol, Shimaa M. Motawei, Eman Hamza

**Affiliations:** 1 Pharmacology and Toxicology Department, Faculty of Pharmacy, Ahram Canadian University, Giza, Egypt; 2 Department of Pharmaceutical Sciences, College of Pharmacy, Princess Nourah Bint Abdulrahman University, Riyadh, Saudi Arabia; 3 Department of Anatomy and Physiology, College of Medicine, Imam Mohammad Ibn Saud Islamic University (IMSIU), Riyadh, Saudi Arabia; 4 Pharmacology and Toxicology Department, Faculty of Pharmacy, October 6 University, Giza, Egypt; 5 Biochemistry Department, Faculty of Pharmacy, Delta University for Science and Technology, Gamasa, Egypt; 6 Clinical Pharmacy Department, Faculty of Pharmacy, Misr International University (MIU), Cairo, Egypt; 7 Department of Clinical Pharmacy and Pharmacy Practice, Faculty of Pharmacy, Ahram Canadian University, 6th of October City, Egypt; 8 Department of Pharmacology and Toxicology, Faculty of Pharmacy, Mansoura National University, Gamasa, Egypt; 9 Biochemistry Department, Faculty of Pharmacy, Ahram Canadian University, 6th of October City, Egypt; 10 Department of Clinical Pharmacy, College of Health Sciences, Al-Rayan Colleges, Madinah, Saudi Arabia; 11 Nursing Department, Al Rayan National College of Health Sciences and Nursing, Madinah, Saudi Arabia; 12 Microbiology and Immunology Department, Faculty of Pharmacy, Ahram Canadian University, Giza, Egypt; 13 Department of Basic Medical Sciences, College of Medicine, AlMaarefa University, Riyadh, Saudi Arabia; 14 Research Center, Deanship of Scientific Research and Post-Graduate Studies, AlMaarefa University, Riyadh, Saudi Arabia; 15 Department of Human Anatomy and Embryology, Faculty of Medicine, Mansoura University, Mansoura, Egypt; 16 Preclinical Medical Department, College of Medicine and Dentistry, Riyadh Elm University (REU), Riyadh, Saudi Arabia; 17 Physiology Department, Faculty of Medicine, Benha University, Benha, Egypt; 18 Department of Anatomy and Embryology, Mansoura Faculty of Medicine, Mansoura University, Mansoura, Egypt; 19 Department of Forensic Medicine and Clinical Toxicology, Faculty of Medicine, Mansoura University, Mansoura, Egypt; 20 Department of Medical Biochemistry and Molecular Biology, Faculty of Medicine, Mansoura University, Mansoura, Egypt

**Keywords:** ATF4, buspirone, CHOP, cyclophosphamide, ER, miR-205/EGLN2, nephrotoxicity

## Abstract

**Background:**

Although microRNAs have been investigated, the reno-protective role of microRNA-205 (miR-205) in cyclophosphamide (CPA)-induced nephrotoxicity has not yet been explored. Therefore, this study evaluated the influence of buspirone on miR-205 and its downstream target EGLN2, focusing on oxidative and ER stress as key drivers of renal injury. This represents a significant gap in nephrotoxicology and epigenetic Toxicology research.

**Methods:**

Animals were randomly allocated among four groups (n = 8), including the normal control group and the CPA group that received one intraperitoneal injection of CPA at 200 mg/kg on day 7. Buspirone at 5 or 10 mg/kg per day was administered by oral gavage for ten consecutive days, whereas CPA injected on the seventh day. At experimental completion, blood samples and renal tissues were subjected to analysis using biochemical, histopathological, immunohistochemical, Western blotting, and qRT-PCR techniques.

**Results:**

CPA induced marked renal dysfunction, reflected by elevated SCr, BUN, NGAL, and KIM-1, with evident histopathological damage. This was accompanied by miR-205 suppression, EGLN2 upregulation, impaired Nrf2 signaling, increased PC levels, and stimulation of PERK/eIF2α/ATF4/CHOP-linked ER stress signaling (*p* < 0.01). Concurrently, activation of JNK1, Fos, and NF-κB increased inflammatory cytokines, and initiated caspase-dependent apoptotic signaling (*p* < 0.01). Buspirone pretreatment mitigated these alterations, accompanied by increased miR-205 expression, reduced EGLN2 expression, enhanced Nrf2-associated antioxidant defenses, and attenuation of ER stress, inflammation, and apoptosis, thereby preserving renal function and tissue integrity.

**Conclusion:**

These findings highlight the preventive renoprotective potential of buspirone against CPA-induced nephrotoxicity and underscore the relevance of miR-205/EGLN2 signaling in renal stress responses. Future toxicokinetic and toxicodynamic studies should evaluate buspirone’s safety in tumor treatment.

## 1 Introduction

Cyclophosphamide (CPA) is widely utilized in oncology and autoimmune disease management because of its potent cytotoxic and immunosuppressive properties (Gu and Samarneh, 2024; El-Dessouki et al., 2026a). Despite its therapeutic effectiveness, its clinical use is frequently accompanied by adverse effects involving multiple organs, among which renal toxicity represents a significant limitation and a major manifestation of drug-induced organ injury (Hałka et al., 2022). It is metabolized into reactive intermediates that disrupt cellular homeostasis, leading to structural and functional renal damage (Alruhaimi et al., 2025). Both experimental and clinical studies have shown that CPA induces tubular injury, inflammatory infiltration, and impaired renal filtration. Therefore, identifying effective pharmacological strategies to attenuate CPA-induced nephrotoxicity remains a key focus in experimental pharmacology and toxicology research on drug-induced renal injury (Alsaidi et al., 2022).

The mechanisms underlying CPA-associated kidney damage are multifactorial and arise from crosstalk among several cellular stress pathways. Evidence indicates that redox imbalance, inflammatory signaling, and apoptotic activation play central roles in nephrotoxicity progression, reflecting underlying toxicodynamic mechanisms (Alghamdi et al., 2024; Saleh et al., 2026). Beyond redox imbalance, endoplasmic reticulum (ER) stress signaling is also recognized for its contribution to the development of renal injury (Cao et al., 2022). Persistent activation of unfolded protein response pathways can drive apoptosis when cellular adaptive responses fail to restore protein-folding homeostasis (Xie et al., 2023).

In recent years, small regulatory RNAs, particularly microRNAs, have become recognized as major controllers of gene activity across physiological regulation and disease-related processes. Among these molecules, microRNA-205 has gained attention for its role in modulating cellular survival, oxidative injury and inflammatory signaling (Gu et al., 2025; Oltra et al., 2025). Altered miR-205 expression has been observed in several pathological conditions, including hepatitis and cardiotoxicity (Romitan et al., 2024; XIAO et al., 2024). Notably, miR-205 targets EGLN2, a prolyl hydroxylase involved in oxygen sensing and cellular stress regulation. Dysregulation of the miR-205/EGLN2 axis has been linked to intensified redox injury with stimulation of stress-related pathways (Zheng et al., 2022; Shafik et al., 2023). Thus, targeting this pathway could provide a useful approach for limiting tissue damage caused by toxic insults, while supporting its relevance in mechanistic toxicology research.

Buspirone is primarily known as an anxiolytic agent acting as a partial agonist of serotonin receptors. However, emerging evidence suggests that several compounds originally developed for neurological applications may exert protective effects against tissue injury through modulation of cellular stress pathways (Fagan and Baldwin, 2023; Jalali et al., 2024). Recent experimental evidence suggests that certain pharmacological agents can regulate antioxidant defenses, suppress inflammatory signaling, and inhibit apoptosis in various experimental models (Thomas Broome and Castorina, 2022; Althagafy et al., 2023). Despite substantial characterization of buspirone’s neuropharmacological profile, its potential protective role in renal injury and its influence on molecular pathways involved in nephrotoxicity remain poorly understood, warranting further mechanistic toxicological profiling of drug-induced renal injury.

Given the close mechanistic crosstalk among oxidative stress, ER stress, inflammatory signaling, and apoptotic pathways during toxicant-induced renal damage, targeting upstream regulatory pathways may offer an effective renoprotective strategy (Wu et al., 2023; Kandel et al., 2025). Accordingly, the current investigation examined whether buspirone could protect against CPA-evoked nephrotoxicity while exploring the underlying molecular mechanisms. Emphasis was placed on the miR-205/EGLN2 axis and its association with Nrf2-mediated antioxidant signaling, PERK/ATF4/CHOP-driven ER stress, NF-κB-mediated inflammation, and apoptosis. Collectively, the study provides integrated molecular insight into the association between miR-205/EGLN2 signaling and buspirone-associated protection against CPA-induced kidney damage.

## 2 Materials and methods

### 2.1 Drugs and chemicals

Cyclophosphamide was utilized as a preparation containing 200 mg per vial and was obtained from Baxter International Inc., based in the United States. Buspirone, sourced from SmithKline Beecham, located in the United Kingdom, was freshly dissolved in distilled water prior to administration. Analytical procedures and biochemical measurements were performed using commercially available assay kits and specific primary antibodies obtained from certified suppliers, with strict adherence to the manufacturers’ recommended protocols. All other reagents used throughout the experimental procedures met analytical-grade specifications.

### 2.2 Animals

Male Wistar rats, 8 weeks of age and weighing 180 ± 20 g, were obtained from the Animal House of Ahram Canadian University. The animals underwent 1 week of acclimatization under controlled laboratory settings maintained at 24 °C and 55% relative humidity. Throughout the experiment, rats were kept in standard bedded cages and environmental enrichment, fed commercial pelleted chow, and provided free access to drinking water. Eight animals were included in each group based on the sample sizes used in comparable previously published experimental studies and consistent with the reduction principle of the 3Rs in animal research. Eligibility was restricted to healthy male Wistar rats within the predefined age and weight ranges.

Following acclimatization, each animal was assigned a unique identification number and distributed equally among the four experimental groups through a computer-generated randomization sequence (1:1:1:1). Investigators responsible for outcome measurements remained blinded to group assignment, although personnel responsible for administering the different interventions were aware of group assignment. Animals were monitored daily for general appearance, activity, food and water intake, and signs of distress. No animal was removed from the study, no dataset was omitted from the final analysis, and the experimental period was completed without unexpected adverse events or mortality. All animal procedures followed institutional ethical requirements, the NRC Guide for the Care and Use of Laboratory Animals, eighth edition (2011), and ARRIVE 2.0 reporting standards. The protocol was finalized before experimentation and received approval from the Faculty of Pharmacy Research Ethics Committee, Ahram Canadian University (REC0726).

### 2.3 Experimental design

**FIGURE 1:**
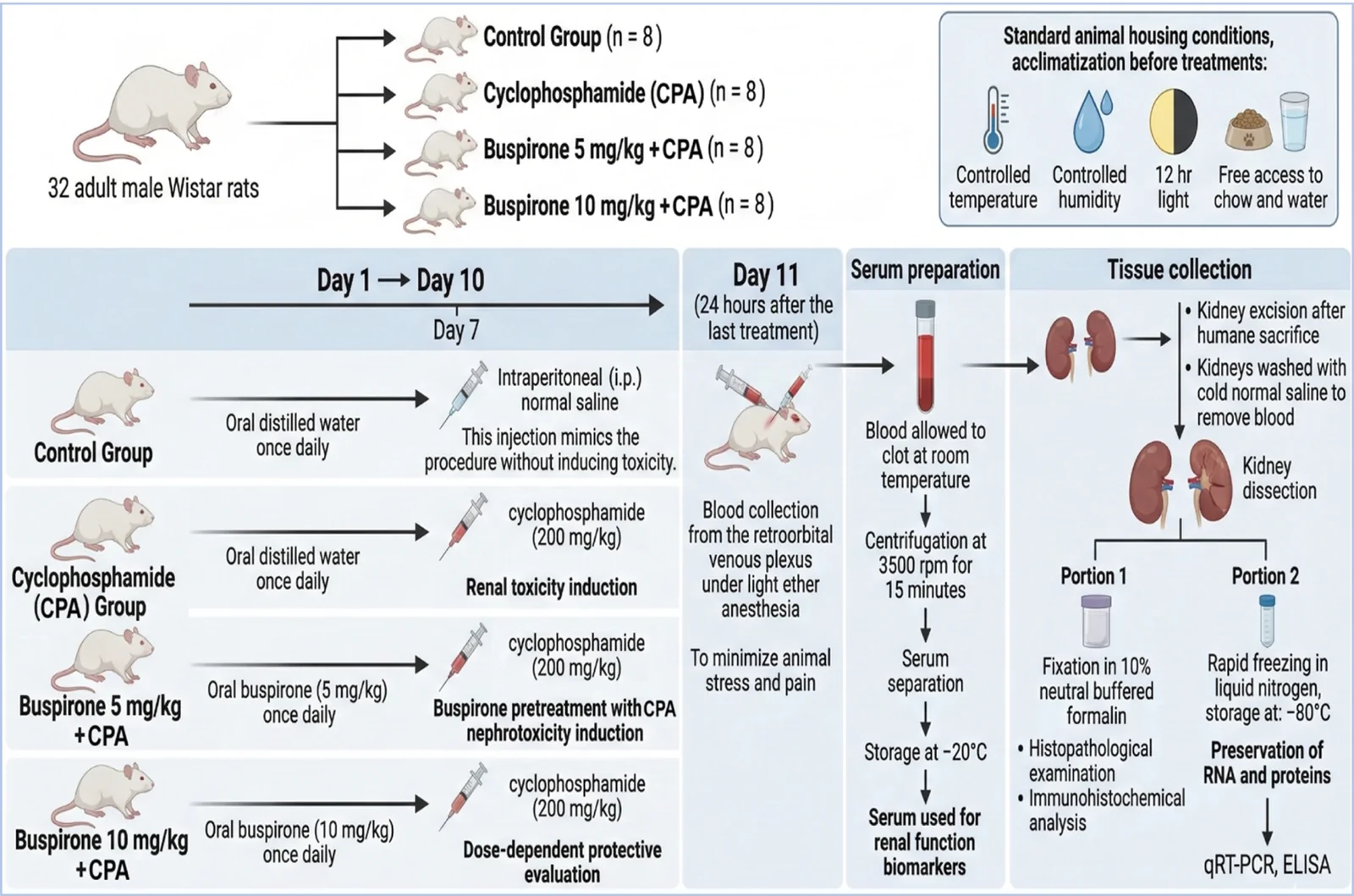
Study design. Male Wistar rats were divided into four groups (n = 8/group). Control rats received distilled water for 10 days, while CPA rats received distilled water and a single CPA injection (200 mg/kg, i. p.) on day 7. Buspirone was given orally at 5 or 10 mg/kg/day for 10 days, with CPA.

Figure 1 summarizes the experimental protocol. A total of 32 male Wistar rats were randomly assigned to four equal experimental groups (n = 8). The control animals were administered distilled water by oral gavage throughout ten consecutive days and were given one intraperitoneal injection of saline on the seventh day. In contrast, the CPA group received distilled water for the same duration, followed by one intraperitoneal injection of CPA at 200 mg/kg on day 7 (El-Dessouki et al., 2026b). In the buspirone-pretreated groups, animals received buspirone orally at 5 or 10 mg/kg/day for 10 consecutive days, with CPA administered intraperitoneally on day 7 (Khalaf et al., 2026). This preventive peri-exposure regimen was selected based on established CPA-toxicity protocols to ensure adequate buspirone administration before the nephrotoxic challenge and during the early progression of renal injury (Rabie et al., 2023; Hussein et al., 2024). At experiment termination (day 11), rats were euthanized, and blood samples together with renal tissues were collected for subsequent analyses.

### 2.4 Sample collection and preparation

On day 11, a single blood sample was withdrawn from the retro-orbital plexus under diethyl ether anesthesia immediately before euthanasia (Noorwali et al., 2024). The collected blood was kept undisturbed at room temperature to allow coagulation, followed by centrifugation at 3,500 rpm for 15 min for serum separation. Serum aliquots were then maintained at −20 °C until measurement of renal function markers, including SCr and BUN. Following blood collection, animals were euthanized, and the kidneys were excised, rinsed using physiological saline, then sectioned. A representative renal tissue portion was fixed in 10% neutral-buffered formalin for histopathological and immunohistochemical evaluation, whereas the residual tissue was snap-frozen in liquid nitrogen and kept at −80 °C for later molecular analyses. Investigators performing histopathological scoring, immunohistochemical quantification, Western blot densitometry, qRT-PCR analysis, and statistical evaluation were blinded to group allocation through the use of coded samples and datasets. Group identities were disclosed only after completion of the primary analyses.

### 2.5 Assessment of serum renal biomarkers

Kidney function indices were quantified using standardized assay kits. BUN levels were assayed using a rat-specific ELISA kit obtained from Bioassay Technology Laboratory (E−0776-Ra). SCr concentration was measured using an assay kit supplied by Crystal Chem (CC-80340). Additionally, KIM-1 was measured using a rat ELISA kit obtained from Bioassay Technology Laboratory (E−3363-Ra), whereas NGAL was determined with a CUSABIO sandwich ELISA kit (CSB-E−09409r). All analytical procedures followed manufacturers’ protocols (Raslan et al., 2025).

### 2.6 Assessment of renal oxidative stress markers

The renal oxidative profile was examined by measuring markers of oxidative injury and endogenous antioxidant defenses. Protein carbonyl content (PC) and Keap1 were evaluated as indicators of oxidative damage, while HO-1 and SOD were measured as key antioxidant markers. Quantification was performed using commercially available ELISA kits, including PC (ELK Biotechnology, ELK9078), Keap1 (ELK Biotechnology, ELK9260), HO-1 (CUSABIO, CSB-E08267r), and SOD (BT-Lab, E1444Ra). All measurements followed manufacturers’ protocols (El-Dessouki et al., 2025b; Saleh et al., 2025a).

### 2.7 Assessment of inflammatory cytokines

Tissue concentrations of TNF-α, IL-1β, and IL-6 were measured with commercially available ELISA kits. Specifically, TNF-α was determined using a kit from R&D Systems (RT-A-001), IL-1β with an assay supplied by Cloud-Clone Corp (SEA-563-Ra), and IL-6 levels were evaluated with a kit provided by Elabscience (ELR-0015). All analytical procedures followed manufacturers’ protocols (El-Dessouki et al., 2025a; Ibrahim et al., 2025).

### 2.8 Evaluation of apoptosis-related markers

Apoptotic signaling within kidney tissue was evaluated through quantification of caspase-12 and caspase-3 expression. Caspase-12 concentrations were determined employing a commercial kit supplied by ELK Biotechnology (ELK9553), whereas caspase-3 levels were measured using an assay kit obtained from Elabscience (ELR-0160). All analytical procedures followed manufacturers’ protocols (Antar et al., 2024; El-Dessouki et al., 2025c).

### 2.9 Histopathology and immunohistochemistry (IHC)

Renal tissue specimens were immediately excised, cleared of residual blood with chilled isotonic saline, and fixed in 10% neutral-buffered formalin for 24–48 h. After fixation, specimens underwent sequential ethanol dehydration, clearing, paraffin wax embedding, and sectioning into 4–5 µm-thick slices. Four sections from each kidney specimen were stained with hematoxylin and eosin (H&E), then examined under light microscopy. The examination was conducted by an independent expert pathologist blinded to group allocation. Renal histopathological lesions were evaluated using the semi-quantitative multiparametric scoring approach shown in Table 2, which reflects the extent of tubular vacuolar degeneration, perivascular edema, inflammatory cell infiltration, and vascular alterations. Scores ranged from 0 to 3, representing absent, mild, moderate, and severe histopathological alterations, respectively, with higher scores indicating greater renal structural injury. Representative photomicrographs were captured using scale bars of 25 and 50 μm (Al-Karmalawy et al., 2024; Tawfik et al., 2024).

Immunohistochemical detection of caspase-9, NF-κB p65, and Nrf2 was performed using paraffin-embedded renal tissue sections prepared at 5 µm thickness. Slides were initially deparaffinized and rehydrated, then subjected to microwave-assisted antigen retrieval in citrate buffer (pH 6.0). Endogenous peroxidase activity was inhibited by incubation in 3% hydrogen peroxide for 10 min. Thereafter, tissue sections were incubated overnight at 4 °C with the selected primary antibodies, including rabbit anti-Nrf2 (1:100–1:200), rabbit anti-NF-κB p65 (1:200), and rabbit anti-caspase-9 (1:150). The following day, sections were exposed to a biotin-conjugated secondary antibody coupled with streptavidin–horseradish peroxidase. Immunoreactive complexes were developed with 3,3′-diaminobenzidine (DAB), and nuclei were counterstained with hematoxylin. The stained slides were inspected using light microscopy (scale reference = 25 µm), and immunopositive areas were quantified using ImageJ software. Representative micrographs were obtained to document the expression patterns of the investigated markers (El-Gamil et al., 2024; Hefny et al., 2024).

### 2.10 Western blot

Renal endoplasmic reticulum stress was assessed by measuring ATF4 and CHOP protein expression. Proteins were extracted from renal tissue homogenates prepared in chilled RIPA buffer supplemented with protease and phosphatase inhibitors (Thermo Fisher Scientific). Homogenates were centrifuged at 12,000 × g for 20 min at 4 °C, after which the clarified supernatants were collected for protein assessment. Protein concentration was quantified with a bicinchoninic acid (BCA) assay kit (Thermo Fisher Scientific). Equal protein loads (30 µg) were resolved using 10%–12% SDS-polyacrylamide gel electrophoresis and transferred onto PVDF membranes (Millipore). Afterward, membranes were blocked for 1 h at room temperature in either 5% nonfat dry milk or 5% bovine serum albumin dissolved in Tris-buffered saline containing 0.1% Tween-20 (TBST). Primary antibodies against ATF4 (Cat. No. 11816, Cell Signaling Technology) and CHOP (Cat. No. 2896, Cell Signaling Technology), both validated for rat samples, were incubated overnight at 4 °C (El-Dessouki et al., 2026a). Following washing with TBST, the membranes were exposed for 1 h at room temperature to suitable horseradish peroxidase (HRP)-conjugated secondary antibodies, including goat anti-rabbit IgG for ATF4 and goat anti-mouse IgG for CHOP (Thermo Fisher Scientific; 1:5,000). Bands were detected with enhanced chemiluminescence (ECL) reagents and captured using a chemiluminescence imaging platform. Densitometric analysis was performed with ImageJ software, and protein expression values were normalized to β-actin (Cat. No. 3700, Cell Signaling Technology), which was used as the internal loading control (Saleh et al., 2025b).

### 2.11 Quantitative real-time polymerase chain reaction (qRT-PCR)

qRT-PCR analysis enabled the assessment of miR-205 expression as well as transcript levels of EGLN2, PERK, eIF2α, GADD34, JNK1, and Fos. Total RNA, including the small RNA fraction, was isolated from renal tissue with TRIzol reagent following the supplier’s protocol. RNA quantity and quality were evaluated spectrophotometrically through measurement of 260-nm absorbance together with the A260/A280 value, which was recorded using a NanoDrop spectrophotometer. RNA samples showing A260/A280 values close to 1.8 were considered suitable for downstream analysis. For miR-205 measurement, cDNA synthesis was performed using a miRNA-specific reverse-transcription procedure, with U6 small nuclear RNA (snRNA) serving as the endogenous normalization control. For mRNA targets, cDNA synthesis was performed from 1 µg total RNA with a Qiagen reverse-transcription PCR kit according to the manufacturer’s protocol, and β-actin served as the reference gene. Gene expression was measured with a Rotor-Gene Q system and Maxima SYBR Green/Fluorescein qPCR Master Mix (El-Dessouki et al., 2024). Each 25-µL reaction contained 12.5 µL SYBR Green master mix, 2 µL forward primer, 2 µL reverse primer, 5.5 µL nuclease-free water, and 3 µL cDNA template. Reactions were run in triplicate for each biological sample. The cycling protocol included initial enzyme activation at 95 °C for 10 min, then 45 cycles consisting of 95 °C for 10 s and 60 °C for 15 s, and extension at 72 °C for 15 s. Primer specificity was confirmed by melting-curve assessment, with a single amplification peak indicating specific product formation. Primer sequences and expected amplicon sizes are provided in Table 1. Relative gene expression was determined using the 2^−ΔΔCt^ approach, with miR-205 normalized to U6 and mRNA targets normalized to β-actin (Shaldam et al., 2024).

**TABLE 1:** Primers used for qRT-PCR.

Gene	Description	Primer sequence	Reference sequence	Product size
miR-205	Forward	5′-ATG​GGT​CCT​CCT​GTC​CTT​CAT-3′	NR_031920.1	79
	Reverse	5′-ATG​CCT​CCT​GAG​CTT​CAC​TCC-3′		
U6	Forward	5′-CTC​GCT​TCG​GCA​GCA​CAT​ATA​CTA-3′	XR_005498700.1	86
	Reverse	5′-ACG​AAT​TTG​CGT​GTC​ATC​CTT​GCG-3′		
EGLN2	Forward	5′-CAC​AGC​CAG​CTA​CAC​CTA​CC-3′	NM_001004083.1	222
	Reverse	5′-ACA​TAG​GCC​ACC​AGC​CAT​AC-3′		
PERK (Eif2ak3)	Forward	5′-GCT​CAA​TGG​GCG​ATG​TCT​GTA-3′	NM_031599.2	329
	Reverse	5′-AAT​GCC​GTA​TCC​GAT​GTG​GG-3′		
eIF2α (Eif2s1)	Forward	5′-GCT​CCA​CCC​AGG​TAT​GTG​AT-3′	NM_019356.1	307
	Reverse	5′-CCA​GAA​AGG​GCT​GCT​TCG​TA-3′		
Ppp1r15a (GADD34)	Forward	5′-GTT​TGC​ACG​AGA​TCG​AAG​CC-3′	NM_133546.3	152
	Reverse	5′-CAG​GCA​GTG​GAA​GAG​ACG​AG-3′		
JNK1 (Mapk8)	Forward	5′-TCA​GGA​GGT​CCC​AAA​GCC​TA-3′	NM_053829.2	452
	Reverse	5′-CCA​AGG​GGC​TTC​TTC​CTA​CA-3′		
Fos	Forward	5′-CTG​CCT​GCA​AGA​TCC​CCA​AT-3′	NM_022197.2	288
	Reverse	5′-AGG​AAC​CAG​ACA​GGT​CCA​CA-3′		
β-actin	Forward	5′-GGA​GAT​TAC​TGC​CCT​GGC​TC-3′	NM_031144.1	217
	Reverse	5′-AAG​GGT​GTA​AAA​CGC​AGC​TC-3′		

### 2.12 Statistical analysis

Normality was checked with the Shapiro–Wilk test, and equality of variances was examined using the Brown–Forsythe test. Datasets fulfilling parametric assumptions were analyzed by one-way ANOVA followed by Tukey’s multiple-comparisons procedure for endpoint-specific pairwise testing. Ordinal histopathological scores were assessed using the Kruskal–Wallis test, followed by Dunn’s multiple-comparisons test. No overall multiplicity correction was applied across the separate prespecified biochemical and molecular endpoints. Continuous variables are reported as mean ± SEM, whereas histopathological scores are presented as medians with interquartile ranges. A p value below 0.05 was considered statistically significant.

## 3 Results

### 3.1 Buspirone ameliorates cyclophosphamide-induced renal functional impairment and tubular injury biomarkers

**FIGURE 2:**
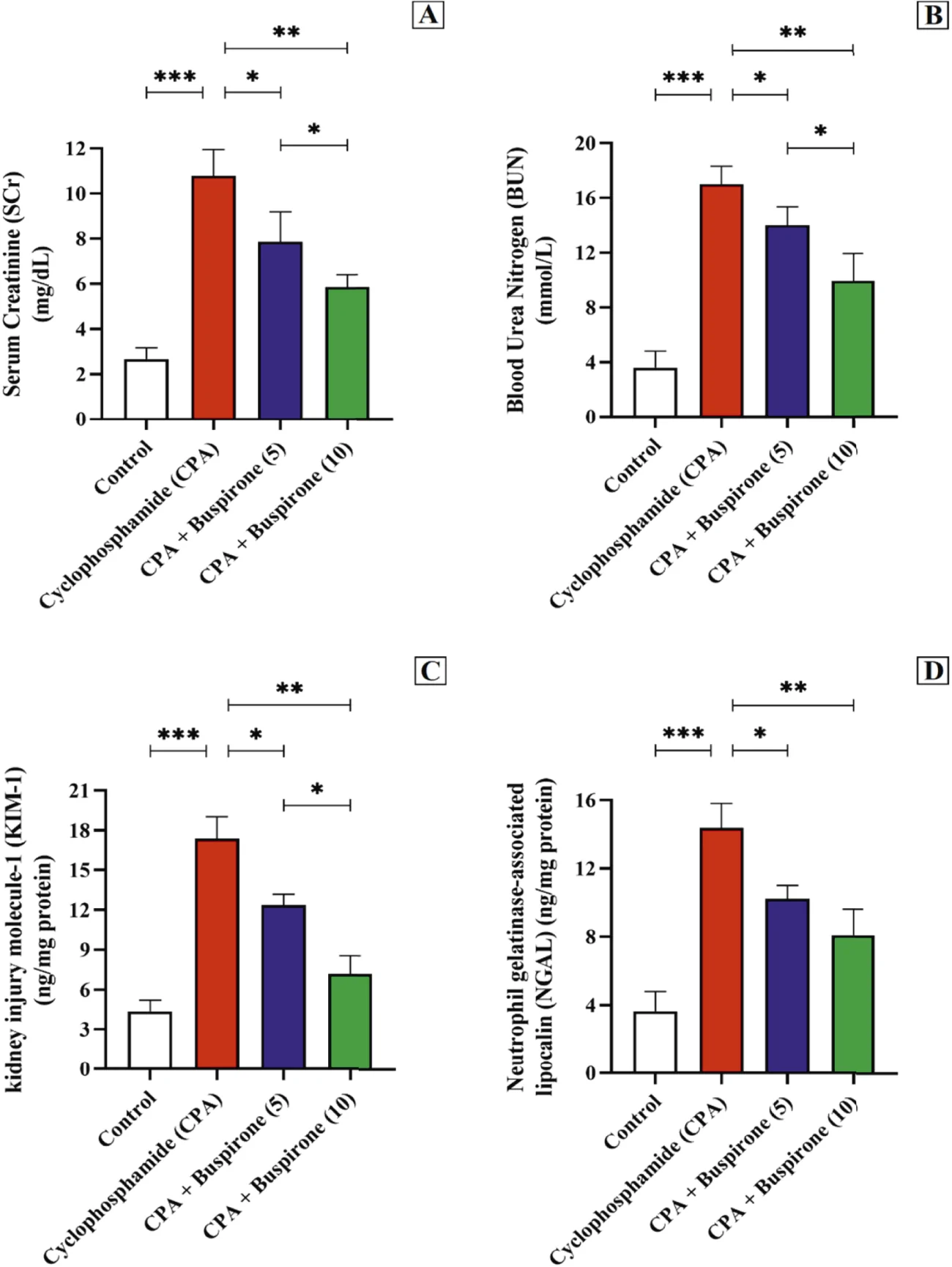
Effect of buspirone on renal function indices and tubular injury markers. **(A)** SCr, **(B)** BUN, **(C)** KIM-1, and **(D)** NGAL. Values indicate the mean ± SEM. ^*^, ^**^, and ^***^ denote p values below 0.05, 0.01, and 0.001, respectively.

To assess CPA-induced renal impairment, SCr, BUN, NGAL, and KIM-1 were determined, providing insight into functional decline and structural damage within renal tissue. CPA administration (200 mg/kg) provoked marked renal deterioration, as reflected by significant elevations in SCr, BUN, KIM-1, and NGAL, which reached 305.26%, 371.94%, 299.54%, and 295.87% of the normal control values (*p* < 0.001). Buspirone given at 5 mg/kg clearly attenuated the CPA-evoked elevations, lowering SCr, BUN, KIM-1, and NGAL levels by 27.18%, 17.60%, 28.78%, and 28.95%, respectively, versus the CPA animals (*p* < 0.05). Buspirone administration at 10 mg/kg produced stronger protection, resulting in reductions reaching 45.64% for SCr, 41.49% in BUN, 58.71% in KIM-1, and 43.70% in NGAL versus the CPA group (*p* < 0.01). Moreover, comparison between the two buspirone doses revealed that animals receiving 10 mg/kg exhibited further declines in SCr, BUN, KIM-1, and NGAL by 25.35%, 29.00%, 42.02%, and 20.76%, respectively, versus the 5 mg/kg dose (*p* < 0.05), supporting a clear dose-related renal protective response (Figures 2A–D).

### 3.2 Buspirone improves cyclophosphamide-induced histopathological alterations in renal tissue

**FIGURE 3:**
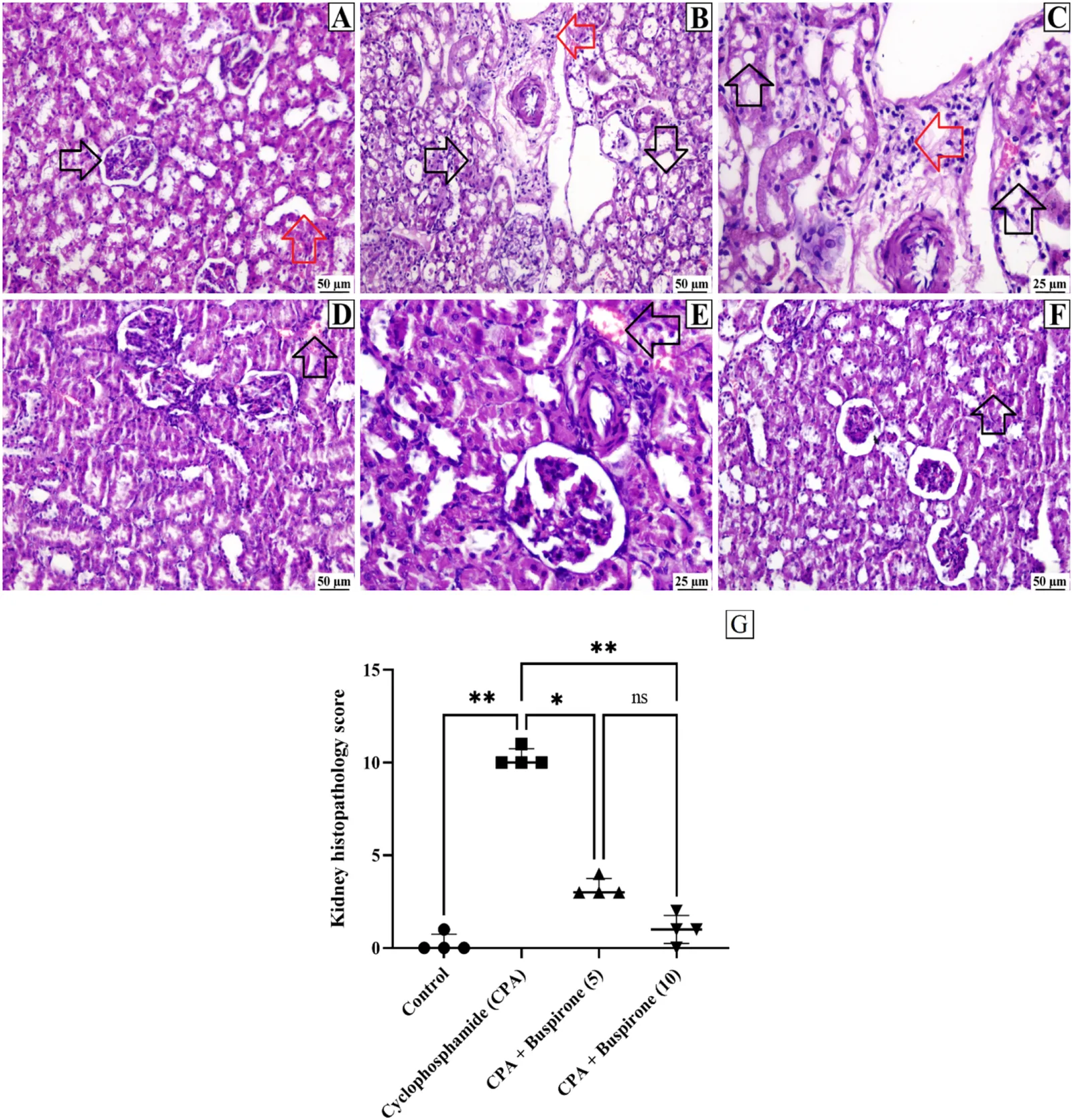
Histological overview of kidney tissue sections. Kidney specimens obtained from the control group exhibited preserved structural organization, including intact renal tubules (red arrow) and glomeruli (black arrow) **(A)**. Inversely, sections from the CPA-treated group revealed prominent pathological alterations, including marked vacuolar degeneration of the tubular epithelium (black arrow) and evident perivascular edema accompanied by infiltration of mononuclear inflammatory cells (red arrow) **(B)**. A higher magnification image **(C)** further illustrates these degenerative and inflammatory changes with greater clarity. In animals receiving buspirone pretreatment at 5 mg/kg, renal sections demonstrated congestion of interstitial blood vessels within the cortical region (black arrow), along with partial attenuation of tissue injury **(D)**. A high-magnification micrograph **(E)** further reveals interstitial vascular congestion. Buspirone administration at 10 mg/kg produced near-normal renal histological features, although mild vascular congestion (red arrow) remained detectable in some areas **(F)**. The semi-quantitative multiparametric histopathological assessment of renal tissue across the studied groups is shown in **(G)**. This grading approach reflects the extent of vacuolar degeneration, perivascular edema, inflammatory cell infiltration, and vascular alterations. Results are displayed as scatter dot plots and summarized as median ± interquartile range. Kruskal–Wallis analysis revealed a marked overall difference among groups (*p < 0.01*).

Kidney sections from control rats showed preserved cortical and medullary architecture (Figure 3A). Conversely, CPA group showed marked histological alterations, primarily tubular damage, notably in the proximal convoluted tubules, which were characterized by severe vacuolar degeneration of tubular epithelium with intratubular cast as well as sporadic areas of coagulative necrosis with pyknotic nuclei. Mild interstitial mononuclear inflammatory cells infiltration was observed in some sections in addition to prominent vascular congestion and perivascular edema with inflammatory infiltrates (Figures 3B,C). In the Buspirone-pretreated group (5 mg/kg) had an ameliorative effect against CPA induced nephrotoxicity. There was mild to moderate degeneration of renal tubule epithelial lining characterized by several morphologic changes, such as cell swelling with or without cytoplasmic vacuolation and pale-staining cytoplasm in addition to vascular congestion (Figures 3D,E). Buspirone at the higher dose (10 mg/kg) preserved renal structure; however, mild congestion and minimal tubular changes were seen in a few individuals (Figure 3F). The semi-quantitative multiparametric histopathological assessment of renal tissue across the studied groups is presented in Figure 3G and Table 2.

**TABLE 2:** Semi-quantitative renal histopathology scoring in rats.

Score	Vacuolar degeneration	Perivascular edema	Inflammatory cells infiltration	Vascular changes
0	No detectable change	No detectable edema	Absent inflammatory cells	No vascular alteration
1	Scattered tubular vacuoles	Limited edema	Sparse inflammatory cells	Minor vascular alteration
2	Moderate tubular epithelial vacuolation	Mild-to-moderate edema	Multifocal	Mild to moderate congestion
3	Extensive tubular epithelial vacuolation	Marked perivascular edema	Confluent inflammatory infiltrates	Mild-to-moderate vascular congestion

### 3.3 Buspirone is associated with restoration of the cyclophosphamide-disrupted miR-205/EGLN2 expression pattern

**FIGURE 4:**
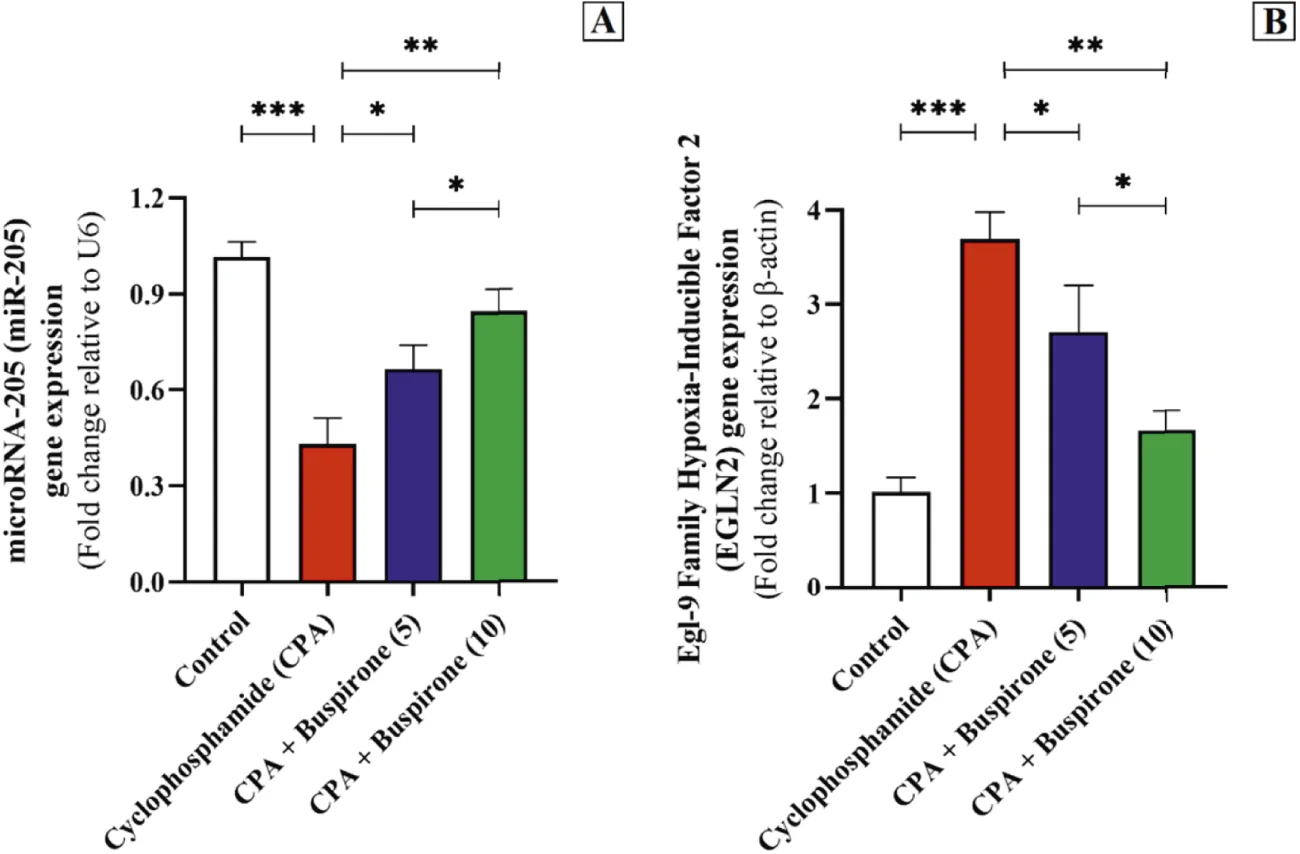
Influence of buspirone on miR-205 and EGLN2 expression. **(A)** miR-205, **(B)** EGLN2. Values indicate the mean ± SEM. ^*^, ^**^, and ^***^ denote p values below 0.05, 0.01, and 0.001, respectively.

To examine alterations in regulatory gene expression linked to renal cellular homeostasis, miR-205 and EGLN2 levels were assessed. CPA administration markedly disrupted this axis, as demonstrated by a significant decline in miR-205 expression to 42.57% of the control value (*p <* 0.001), accompanied by a pronounced elevation in EGLN2 expression to 265.35% (*p <* 0.001). Buspirone pretreatment at 5 mg/kg partially restored the disrupted expression profile, increasing miR-205 expression by 53.49% while reducing EGLN2 expression by 26.83% versus the CPA group (*p <* 0.05). Buspirone administration at 10 mg/kg exerted a stronger regulatory modulation, elevating miR-205 by 95.35% and suppressing EGLN2 by 55.01% (*p <* 0.01). Furthermore, buspirone at 10 mg/kg achieved greater normalization in this expression pattern, with miR-205 further increased by 27.27% and EGLN2 further reduced by 38.52% versus the 5 mg/kg group *(p <* 0.05), showing dose-related normalization of gene expression (Figures 4A,B).

### 3.4 Buspirone restores Nrf2-dependent antioxidant signaling and reduces oxidative protein damage

**FIGURE 5:**
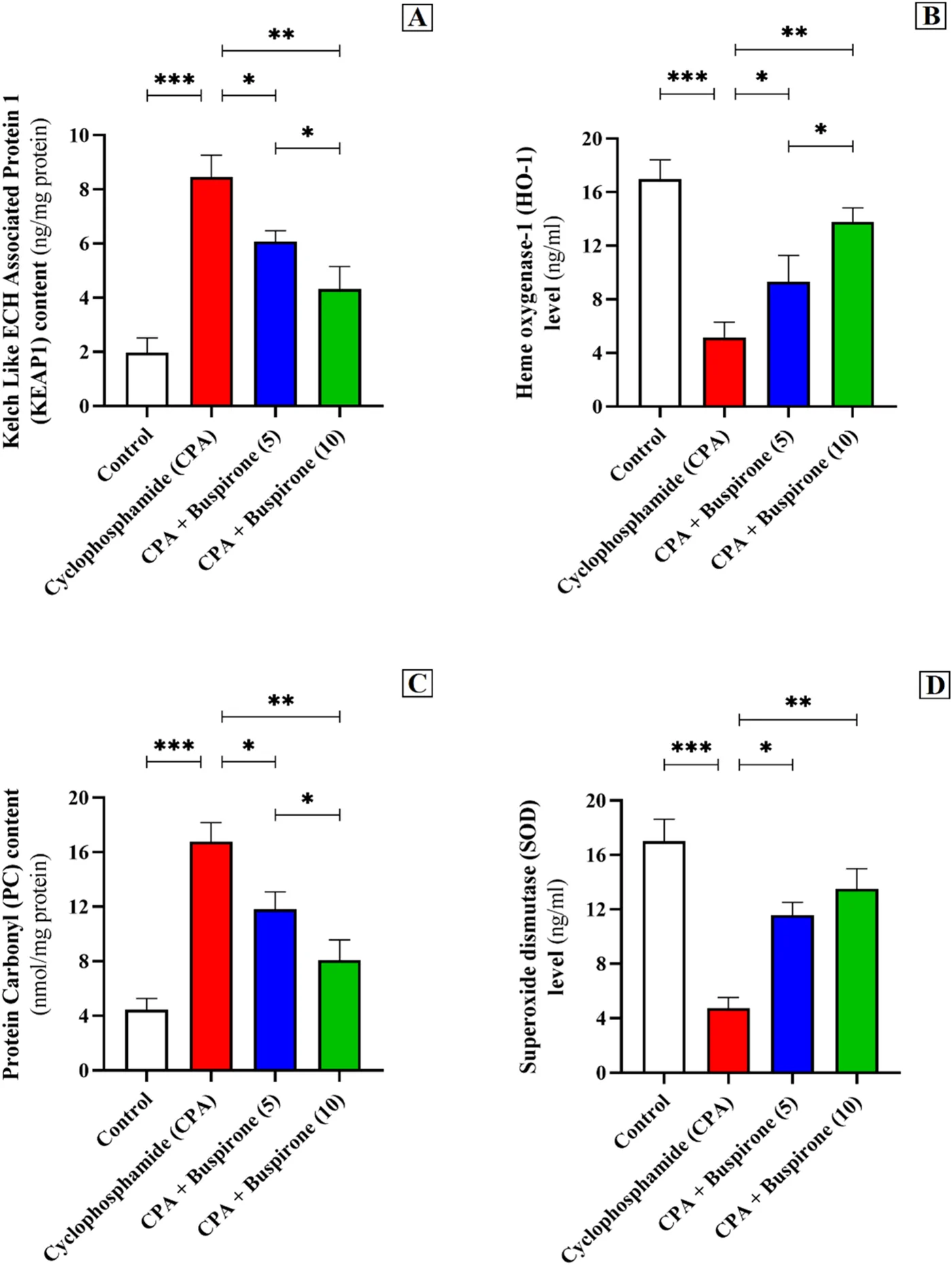
Effect of buspirone on oxidative stress markers and antioxidant defense components. **(A)** Keap1, **(B)** HO-1, **(C)** PC, and **(D)** SOD. Values indicate the mean ± SEM. ^*^, ^**^, and ^***^ denote p values below 0.05, 0.01, and 0.001, respectively.

**FIGURE 6:**
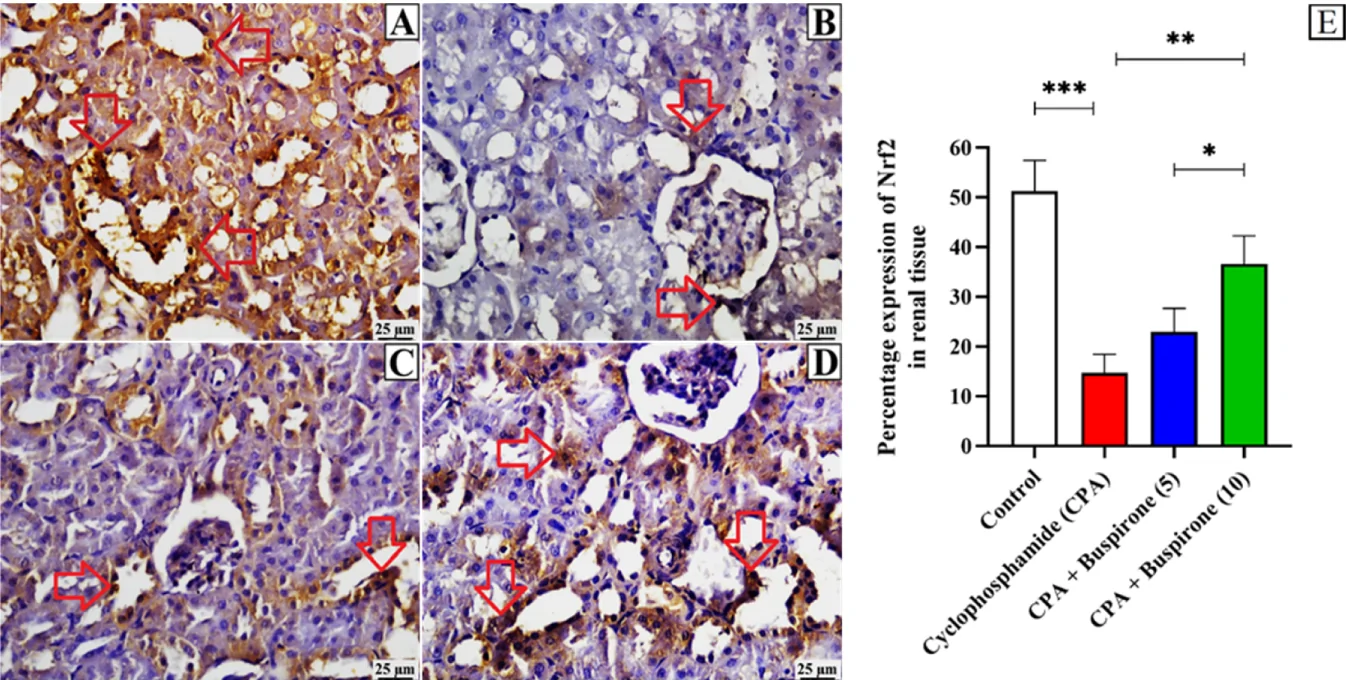
Buspirone regulates renal Nrf2 immunoexpression. Renal sections from control animals showed prominent cytoplasmic Nrf2 staining positivity within tubular cells (arrowhead) **(A)**. CPA administration was associated with a pronounced loss of Nrf2 expression, with sparse immunopositive cells detected (arrowhead) **(B)**. Buspirone at 5 mg/kg induced mild recovery of Nrf2 immunoreactivity (arrowhead) **(C)**. Buspirone at 10 mg/kg produced a more evident improvement, characterized by moderate staining, particularly around vascular regions (arrowhead) **(D)** (scale reference: 25 µm). **(E)** presents the quantitative assessment of renal Nrf2 immunostaining. Values indicate the mean ± SEM. ^*^, ^**^, and ^***^ denote p values below 0.05, 0.01, and 0.001, respectively.

Marked oxidative disruption was evident following CPA exposure, as demonstrated by assessment of Keap1, HO-1, PC, and SOD by ELISA, along with Nrf2 immunoreactivity, collectively reflecting redox imbalance and oxidative protein modification. CPA administration resulted in a substantial rise in Keap1 and PC levels to 331.12% and 279.19%, respectively, accompanied by a pronounced reduction in HO-1 and SOD to 30.27% and 27.78% of control values (*p* < 0.001). Buspirone pretreatment with 5 mg/kg partially reversed the CPA-induced oxidative changes, decreasing Keap1 and PC by 28.28% and 29.59%, respectively, while enhancing HO-1 and SOD by 80.90% and 144.92%, in the same order versus the CPA group (*p* < 0.05). Stronger restoration occurred following buspirone dosing at 10 mg/kg, where Keap1 and protein carbonyls declined by 48.99% and 51.91%, respectively, alongside elevations in HO-1 and SOD reaching 168.23% and 186.02%, respectively (*p* < 0.01). Further comparison between both doses revealed additional reductions in Keap1 and PC by 27.85% and 31.69%, respectively, along with a further increase in HO-1 by 48.28% beyond the response observed with 5 mg/kg (*p* < 0.05), supporting dose-dependent restoration of redox balance, as displayed in Figures 5A–D. To further support the observed antioxidant response, Nrf2 expression was examined in renal tissue. Immunohistochemical staining was performed, and staining intensity was quantitatively assessed, as illustrated in Figures 6A–E.

### 3.5 Buspirone suppresses PERK/eIF2α/ATF4/CHOP-associated endoplasmic reticulum stress signaling

**FIGURE 7:**
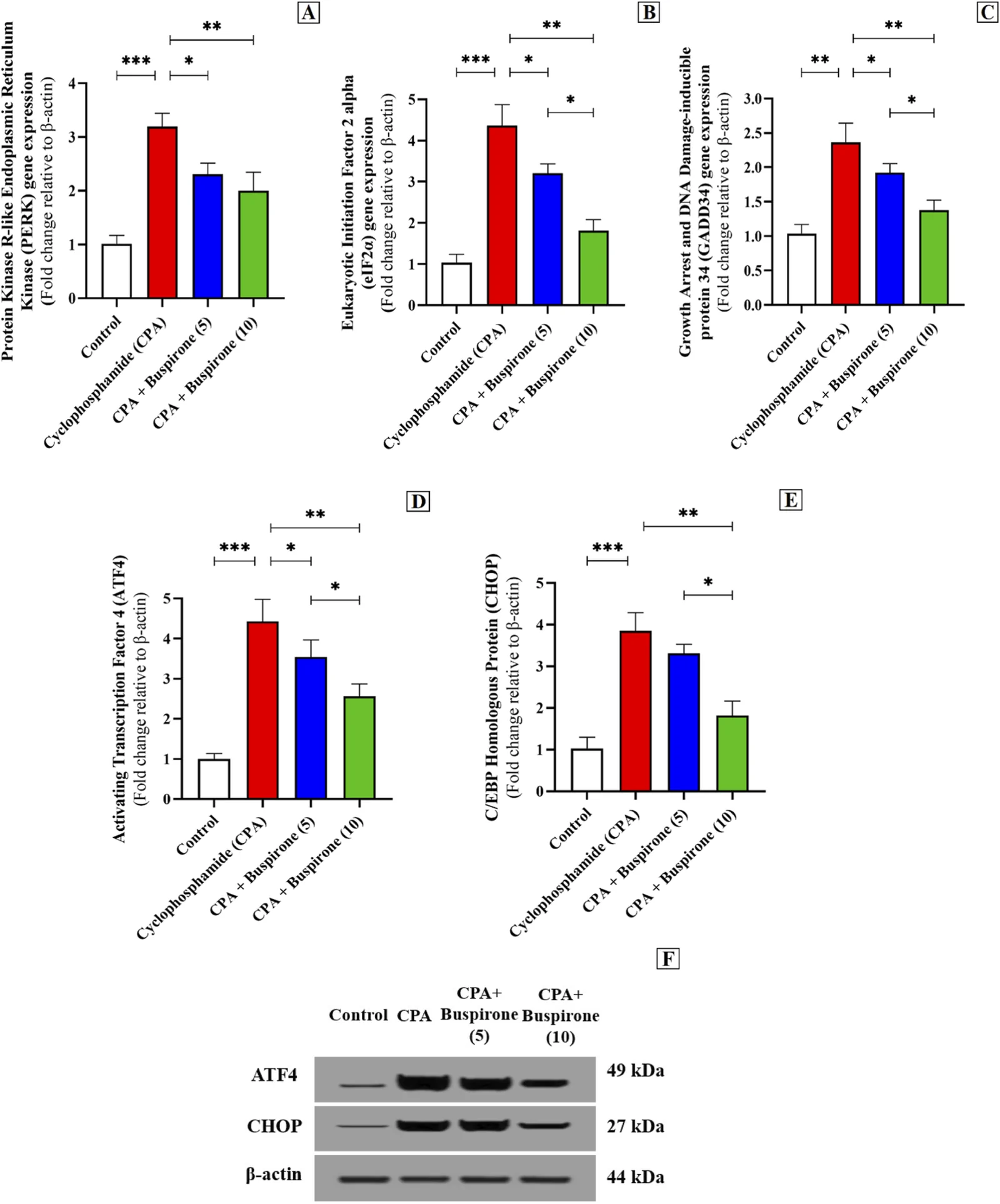
Buspirone modulates stress-related signaling. **(A)** PERK, **(B)** eIF2α, **(C)** ATF4, **(D)** CHOP, **(E)** GADD34, and **(F)** Representative immunoblots of ATF4 and CHOP with their corresponding loading control (β-actin). Values indicate the mean ± SEM. ^*^, ^**^, and ^***^ denote p values below 0.05, 0.01, and 0.001, respectively.

Assessment of endoplasmic reticulum stress pathway activation was conducted by quantifying PERK, eIF2α, and GADD34 gene expression using RT-PCR, while CHOP and ATF4 were evaluated as protein expression using immunoblotting, collectively reflecting impairment of protein-folding homeostasis and engagement of the PERK-linked unfolded protein response pathway. CPA exposure markedly triggered ER dysfunction, demonstrated by marked induction of PERK, eIF2α, GADD34, along with a pronounced increase in ATF4 and CHOP protein expression to 215.84%, 323.30%, 129.13%, 346.46%, and 286.27% of normal values, respectively (*p* < 0.001 for PERK, eIF2α, ATF4, and CHOP; *p <* 0.01 for GADD34). Buspirone at 5 mg/kg attenuated these elevations, reducing PERK, eIF2α, GADD34, and ATF4 by 27.90%, 26.61%, 18.64%, and 20.13%, respectively, versus the CPA group (*p* < 0.05). Buspirone at 10 mg/kg exerted greater inhibitory activity, decreasing PERK, eIF2α, GADD34, ATF4, and CHOP by 37.62%, 58.49%, 41.95%, 42.30%, and 52.86%, respectively (*p* < 0.01). Furthermore, analysis of the two buspirone dose groups revealed that the 10 mg/kg dose caused further decreases in PERK, eIF2α, GADD34, ATF4, and CHOP by 13.48%, 43.44%, 28.65%, 27.76%, and 45.15%, respectively, versus the 5 mg/kg dose (*p* < 0.05) (Figures 7A–F).

### 3.6 Buspirone inhibits JNK-driven stress signaling and NF-κB-mediated inflammatory responses

**FIGURE 8:**
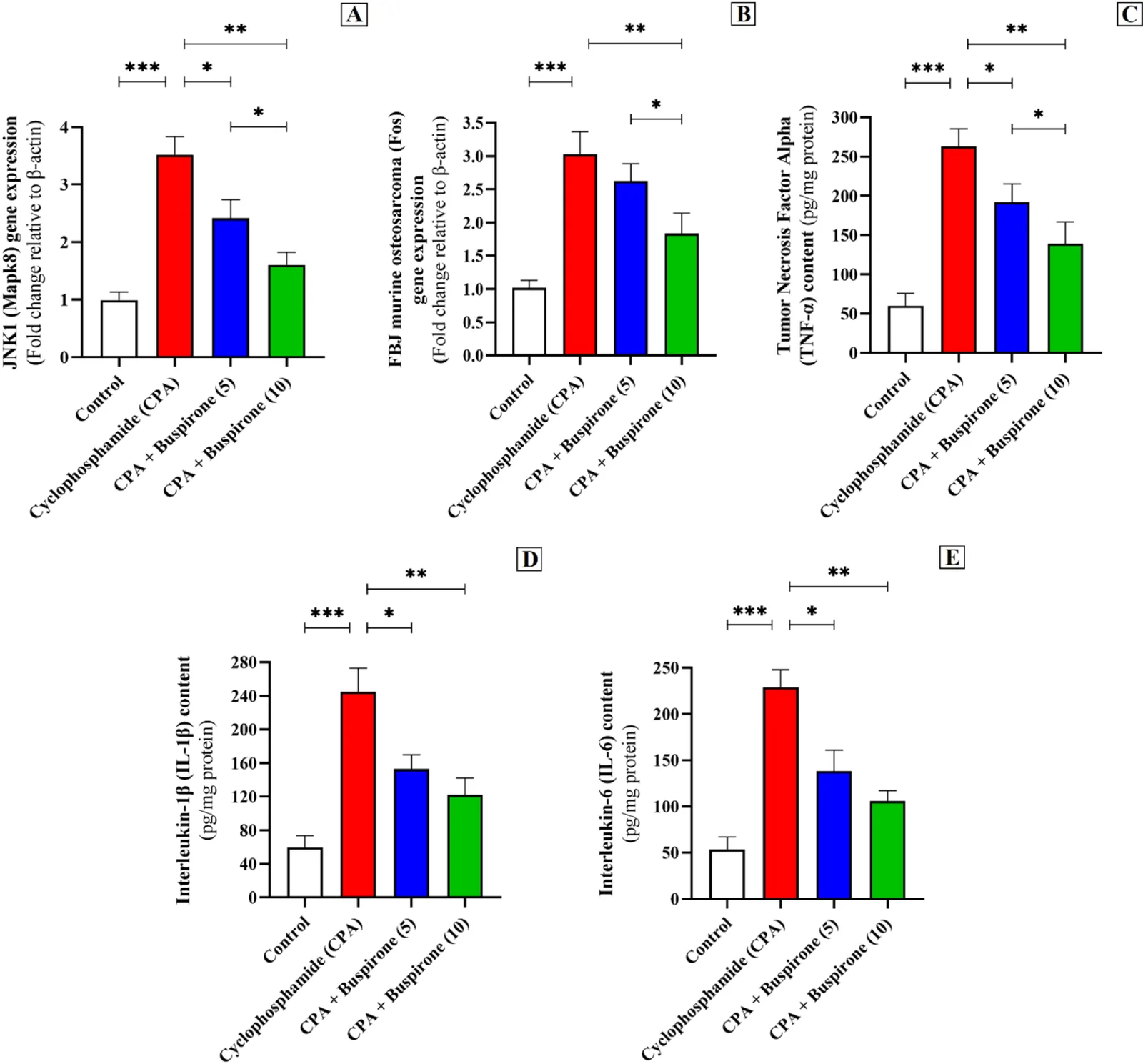
Impact of buspirone on stress-activated signaling and inflammatory mediators. **(A)** JNK1, **(B)** FOS, **(C)** TNF-α, **(D)** IL-1β, and **(E)** IL-6. Values indicate the mean ± SEM. ^*^, ^**^, and ^***^ denote p values below 0.05, 0.01, and 0.001, respectively.

Marked stimulation of stress-responsive and inflammatory signaling routes was observed following CPA exposure, as indicated by elevated expression of JNK and FOS, increased NF-κB p65 immunoreactivity, and heightened TNF-α, IL-1β, and IL-6 concentrations, collectively reflecting intensified inflammatory signaling. CPA administration led to significant increases in JNK, FOS, TNF-α, IL-1β, and IL-6, reaching 255.56%, 199.01%, 336.64%, 313.01%, and 329.19% of normal values (*p* < 0.001). Buspirone pretreatment at 5 mg/kg partially attenuated this response, reducing JNK, TNF-α, IL-1β, and IL-6 levels by 31.53%, 26.99%, 37.71%, and 39.44%, respectively, versus the CPA group (*p* < 0.05), while buspirone administration at 10 mg/kg produced stronger suppression, lowering JNK, FOS, TNF-α, IL-1β, and IL-6 levels by 54.55%, 39.40%, 46.99%, 50.24%, and 53.66%, respectively (*p* < 0.01). Analysis of both doses revealed that the 10 mg/kg dose caused additional decreases in JNK and TNF-α by 33.61% and 27.40%, respectively, versus the 5 mg/kg dose (*p* < 0.05) (Figures 8A–E).

**FIGURE 9:**
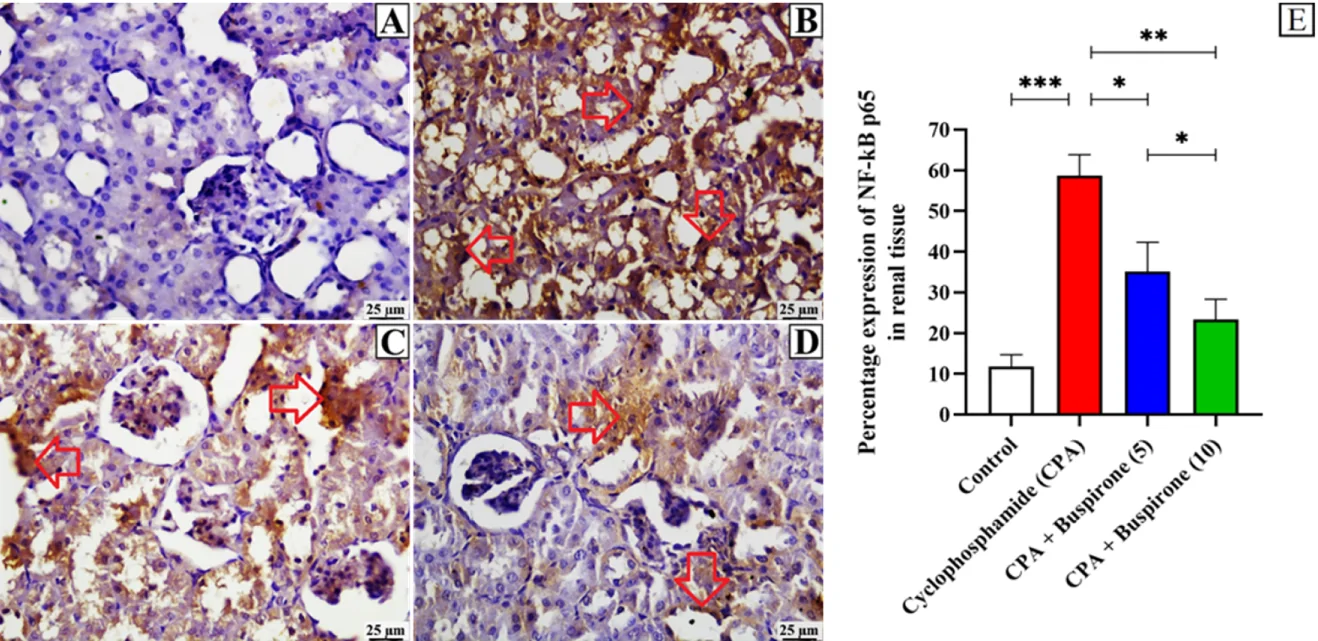
Buspirone-mediated regulation of renal NF-κB p65 immunoexpression. Renal tissues from control rats demonstrated negligible NF-κB p65 immunostaining **(A)**. Conversely, rats administered CPA exhibited pronounced cytoplasmic immunoreactivity within renal tubular epithelial cells (arrowhead) **(B)**. Buspirone at 5 mg/kg induced moderate attenuation of NF-κB p65 staining, predominantly observed as perivascular staining **(C)**. Buspirone at the higher dose (10 mg/kg) markedly weakened NF-κB p65 immunoexpression, with only faint immunostaining detected **(D)** (scale reference: 25 µm). **(E)** Renal NF-κB p65 immunostaining was quantitatively assessed and expressed as mean ± SEM (^*^, ^**^, and ^***^ denote p values below 0.05, 0.01, and 0.001, respectively).

These observations support the inhibitory influence of buspirone on inflammatory mediators; therefore, immunohistochemical evaluation of NF-κB p65 was conducted to further substantiate these findings and visualize its tissue distribution (Figures 9A–E).

### 3.7 Buspirone attenuates ER stress–and mitochondria-associated apoptotic signaling

**FIGURE 10:**
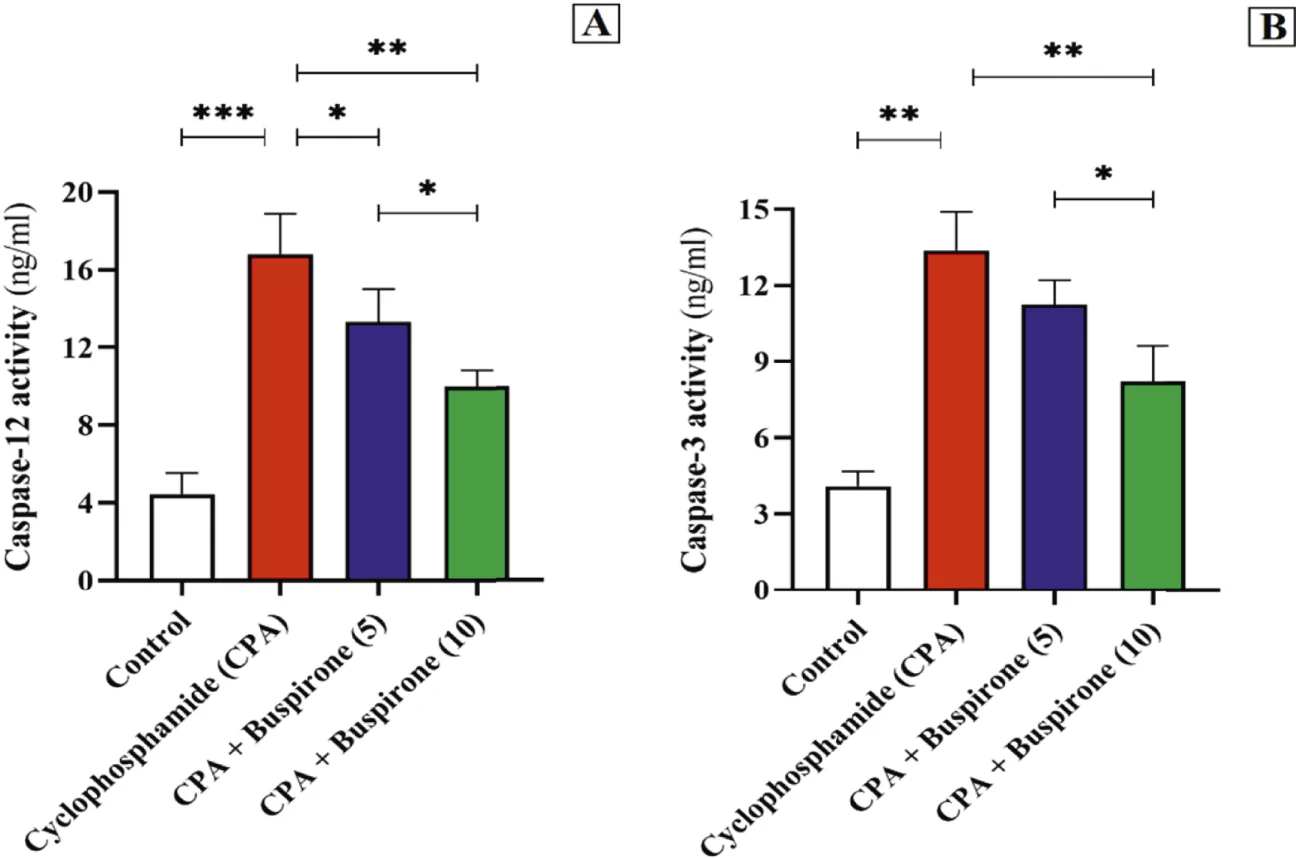
Buspirone regulates apoptotic markers, indicating attenuation of cell death signaling. **(A)** Caspase-12 and **(B)** Caspase-3. Values indicate the mean ± SEM. ^*^, ^**^, and ^***^ denote p values below 0.05, 0.01, and 0.001, respectively.

**FIGURE 11:**
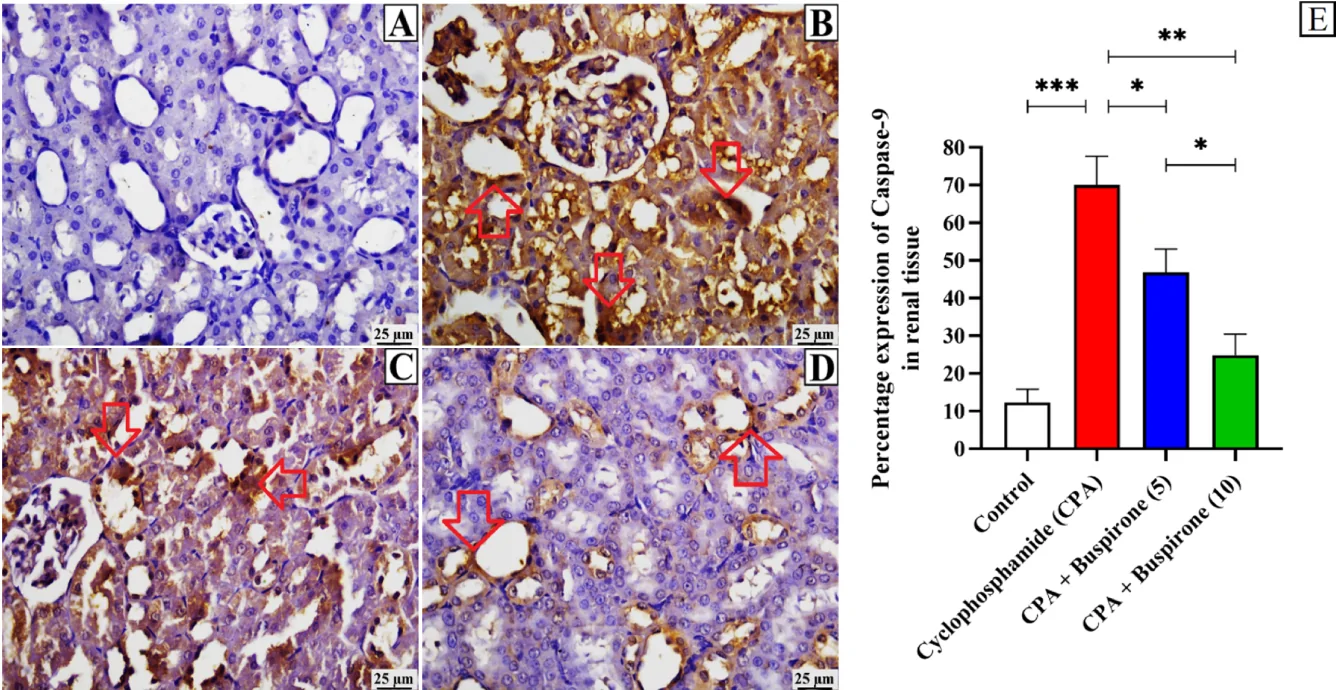
Modulatory effect of buspirone on renal caspase-9 expression. Light microscopic inspection of kidney tissue sections demonstrated an absence of detectable caspase-9 immunoreactivity in the control group **(A)**. Conversely, CPA administration induced a marked elevation in caspase-9 expression, evidenced by intense and widespread cytoplasmic staining within renal tubular epithelial cells (arrowheads) **(B)**. Administration of buspirone at 5 mg/kg partially mitigated this effect, as indicated by moderate immunopositive staining predominantly localized around vascular regions (arrowheads) **(C)**. Notably, buspirone at 10 mg/kg caused a pronounced reduction of caspase-9 expression, with only a limited number of scattered positively stained cells observed (arrowheads) **(D)** (scale reference: 25 µm). **(E)** Panel E presents the numerical assessment of renal caspase-9 immunostaining; values are given as mean ± SEM (^*^, ^**^, and ^***^ denote p values below 0.05, 0.01, and 0.001, respectively).

To assess apoptotic signaling, renal caspase-12 and caspase-3 concentrations were quantified by ELISA, whereas caspase-9 immunoexpression was evaluated by immunohistochemistry. These markers were used to assess ER stress-related apoptosis, intrinsic mitochondrial apoptotic signaling, and activation of downstream executioner caspases. CPA administration markedly triggered apoptotic signaling, as evidenced by a significant increase in caspase-12%–277.53% and caspase-3–227.14% of control values (*p* < 0.001). Buspirone given at 5 mg/kg reduced this apoptotic response, decreasing caspase-12 by 20.71% versus the CP group (*p* < 0.05). Buspirone administration at 10 mg/kg exerted stronger anti-apoptotic activity, reducing caspase-12 and caspase-3 by 40.54% and 38.71%, respectively (*p* < 0.01). Furthermore, analysis of the two dose levels showed that the 10 mg/kg dose induced a further decrease of caspase-12 by 25.00% versus 5 mg/kg dosing (*p* < 0.05) (Figures 10A,B). These results emphasize buspirone’s ability to limit apoptosis through modulation of caspase activity; accordingly, immunohistochemical assessment of caspase-9 was conducted to support the biochemical data and visualize its expression pattern (Figures 11A–E).

## 4 Discussion

Kidney damage caused by cyclophosphamide (CPA) is largely attributed to the generation of toxic metabolites that disrupt cellular homeostasis and trigger multiple stress signaling pathways (Ayza et al., 2022; Oberiukhtin et al., 2026). This research examined whether buspirone could ameliorate CPA-induced renal injury while focusing on the regulatory role of the miR-205/EGLN2 signaling axis and its interaction with antioxidant defense, endoplasmic reticulum stress, inflammatory signaling, and apoptotic mechanisms. The obtained results demonstrated that CPA administration produced profound renal dysfunction, which was manifested by marked increases in SCr and BUN together with elevated levels of NGAL and KIM-1. These biochemical indicators reflect impaired renal filtration and tubular injury, in agreement with data documented in earlier reports (Alruhaimi et al., 2026; El-Dessouki et al., 2026c). Buspirone pretreatment markedly restored these functional parameters, suggesting a protective effect against drug-induced renal impairment. These observations are consistent with previous studies reporting that protective agents targeting intracellular stress pathways can mitigate nephrotoxicity induced by chemotherapeutic drugs, supporting the relevance of this model in evaluating chemotherapy-related organ toxicity (Zaaba et al., 2025; Weldemichael et al., 2026).

Alongside the functional deterioration, CPA exposure resulted in severe structural alterations within renal tissue. Histopathological examination revealed tubular damage characterized by severe vacuolar degeneration of the tubular epithelium with intratubular casts, as well as sporadic areas of coagulative necrosis with pyknotic nuclei, alongside mild interstitial infiltration of mononuclear inflammatory cells. These structural alterations were associated with a marked elevation in the overall kidney damage score, a finding that is consistent with observations reported in previous experimental studies of renal injury (Alaqeel and Al-Hariri, 2023; Mahmood et al., 2025). Conversely, buspirone administration significantly improved renal architecture.

Marked ROS overproduction following CPA exposure can alter redox-sensitive transcriptional control of microRNAs, which likely contributes to the observed suppression of miR-205 expression. Given that miR-205 directly restrains EGLN2 translation, its downregulation removes this inhibitory influence, resulting in pronounced upregulation of EGLN2. Mechanistically, increased EGLN2 expression can potentiate oxidative stress by enhancing pro-oxidant intracellular signaling and impairing mitochondrial efficiency, leading to increased electron leakage and sustained ROS accumulation (Mumby and Adcock, 2022; Shafik et al., 2023). This elevation in ROS not only aggravates oxidative damage but also perpetuates further suppression of miR-205, establishing a self-amplifying loop that reinforces redox imbalance. In parallel, heightened EGLN2 activity may promote cellular susceptibility to stress by facilitating maladaptive metabolic and oxidative responses within renal cells (Alharbi et al., 2024; Almalki and Almujri, 2024; Zhang et al., 2024). Thus, CPA-induced miR-205 suppression drives EGLN2 overexpression, which in turn amplifies oxidative burden and disrupts cellular homeostasis, positioning this axis as a central molecular bridge connecting oxidative damage with renal tissue injury.

Building on this redox imbalance, CPA exposure was associated with a pronounced impairment in renal antioxidant defenses, particularly via disturbance of the Nrf2/Keap1 signaling pathway. The oxidative milieu generated downstream of miR-205/EGLN2 dysregulation likely contributes to suppression of Nrf2 activity, as evidenced by its reduced expression alongside a concomitant increase in Keap1 levels (Dong et al., 2024). Given that Nrf2 is a major transcriptional driver of cellular antioxidant defense, its inhibition compromises the production of key cytoprotective enzymes (Cazalla et al., 2024; Cheng et al., 2024). This antioxidant failure was evident by decreased HO-1 expression and diminished SOD activity, coupled with a marked elevation in PC content, indicating extensive oxidative modification of cellular proteins, which supports findings from previous research (Ince et al., 2024; Shigidi et al., 2026). Taken together, these data indicate that CPA-induced disruption of the miR-205/EGLN2 axis propagates oxidative stress, which in turn impairs Nrf2-dependent antioxidant defenses, thereby exacerbating renal oxidative injury.

Buspirone pretreatment effectively restored the disrupted miR-205/EGLN2 axis, as indicated by increased miR-205 and reduced EGLN2 expression, reflecting reestablishment of post-transcriptional regulation. This normalization likely attenuates the pro-oxidant environment induced by CPA, limiting excessive ROS generation and stabilizing redox balance. In parallel, buspirone enhanced Nrf2 activity, promoting antioxidant gene expression and reducing oxidative damage. The concurrent changes of miR-205/EGLN2 and Nrf2 suggests that buspirone confers renoprotection through coordinated regulation of redox homeostasis at both post-transcriptional and transcriptional levels, consistent with previous reports highlighting these pathways in protection against toxicant-induced renal injury (Shafik et al., 2023; Shi et al., 2025).

The reciprocal alterations in miR-205 and EGLN2 detected in the present study are biologically supported by previous functional evidence demonstrating direct binding of miR-205 to the EGLN2 3′-UTR in renal tubular cells (Muratsu-Ikeda et al., 2012). Moreover, modulation of this axis has previously been associated with Nrf2 activation and ATF4/CHOP suppression in a drug-induced nephrotoxicity model (Shafik et al., 2023). Nevertheless, because miR-205 and EGLN2 were not functionally manipulated in the present model, the observed changes demonstrate an association and do not establish that this axis directly mediates buspirone-induced renoprotection.

Beyond oxidative stress, endoplasmic reticulum (ER) stress represents an additional mechanistic layer contributing to CPA-induced renal injury. The ROS overload together with disrupted redox balance can disrupt protein processing capacity inside the ER, thereby favoring the accumulation of improperly folded proteins and initiating unfolded protein response (UPR) (Casas-Martinez et al., 2024; Dong et al., 2024). CPA markedly activated the PERK signaling cascade, as evidenced by increased expression of PERK and its downstream effector eIF2α, along with upregulation of transcriptional mediators ATF4 and CHOP (Chen et al., 2023; Tóth et al., 2024). The concomitant elevation of GADD34 further supports sustained activation of the PERK-dependent stress pathway. Mechanistically, persistent ER stress under these conditions reflects a failure of adaptive signaling, thereby shifting the UPR from a protective to a maladaptive response that exacerbates cellular dysfunction (Hicks et al., 2023; Casas-Martinez et al., 2024). Importantly, the emergence of ER stress appears to be closely intertwined with oxidative stress, as ROS not only initiate UPR activation but are also further amplified by ER dysfunction, creating a self-propagating cycle of cellular stress (Chen et al., 2023; Xing et al., 2026). These results align with earlier studies identifying ER stress signaling as a major factor in chemotherapy-associated renal toxicity (Shu et al., 2022; Deng et al., 2023; Hu and Chen, 2025).

Buspirone significantly suppressed ER stress signaling, evidenced by decreased PERK, eIF2α, ATF4, CHOP, and GADD34 expression, indicating inhibition of PERK-associated UPR activation. This effect likely reflects restoration of redox balance, reducing ROS-induced protein misfolding and alleviating ER stress triggers. By lowering oxidative stress and stabilizing protein folding, buspirone interrupts the cycle between oxidative damage and ER dysfunction. Thus, UPR modulation contributes to its nephroprotective effect, alongside regulation of the miR-205/EGLN2 axis and Nrf2-mediated antioxidant defense. These observations are consistent with earlier studies showing that attenuation of ER stress and restoration of redox balance are associated with reduced toxicant-induced renal injury (Zhao et al., 2024; Li et al., 2025).

CPA exposure extended beyond oxidative and ER stress to engage stress-activated kinase signaling that critically contributes to renal injury progression. This was demonstrated by marked upregulation of JNK1 and its downstream target FOS, reflecting activation of the JNK/AP-1 axis. At the mechanistic level, accumulated ROS and unresolved ER stress promote activation of upstream kinases such as ASK1, which subsequently phosphorylate and activate JNK1. Once activated, JNK1 translocates to the nucleus and enhances transcriptional activity of AP-1 components, particularly FOS, thereby driving transcriptional activity related to cellular stress responses, inflammatory activation, and cell fate determination (Lin et al., 2023; Kang et al., 2024). Sustained JNK1/FOS signaling is known to shift this response toward a maladaptive state, favoring pro-inflammatory and pro-apoptotic gene expression, a finding that is consistent with earlier reports demonstrating that prolonged JNK/AP-1 activation contributes to driving inflammatory and apoptotic pathways (Zhou et al., 2022; Liu et al., 2024).

Importantly, JNK1 signaling functionally intersects with NF-κB pathways, where JNK1 can enhance NF-κB transcriptional competence either through modulation of co-activators or by influencing upstream signaling nodes that regulate NF-κB activation. This integration amplifies inflammatory output, as reflected by the concurrent elevation of NF-κB p65 together with inflammatory cytokines. These mediators further potentiate JNK activation and oxidative burden, establishing a feed-forward loop that sustains cellular stress and accelerates kidney injury (Alaaeldin et al., 2023; Keçeci et al., 2025). These findings indicate that CPA-induced activation involving the JNK1/NF-κB axis represents an important mechanistic connection between upstream oxidative and ER stress and downstream inflammatory responses, in agreement with earlier investigations showing that JNK–NF-κB interaction contributes to coordinated inflammation under stress conditions (Zhang et al., 2021; Lan et al., 2022; Dong et al., 2024).

Buspirone pretreatment suppressed JNK1 expression and reduced FOS levels, indicating inhibition of stress kinase signaling. This was associated with decreased NF-κB p65 expression and a decline in the inflammatory cytokine profile, suggesting disruption of the stress–inflammation axis. Mechanistically, this effect may be attributed to restoration of redox balance and attenuation of ER stress, thereby limiting activation of stress-responsive kinases and downstream inflammatory pathways. Taken together, the data point to buspirone mediated renal protection, at least partly, by interrupting stress-driven inflammatory signaling, in line with earlier reports describing the anti-inflammatory and cytoprotective actions of buspirone through modulation of stress-associated signaling pathways (Thomas Broome and Castorina, 2022; Khalaf et al., 2026).

The interplay between ER stress, oxidative imbalance, alongside inflammatory signaling ultimately culminates in the activation of apoptotic pathways. CPA exposure markedly upregulated caspase-12, which is commonly associated with ER stress-mediated apoptosis (Khamis et al., 2023; Wang et al., 2024). Additionally, engagement of mitochondria-dependent intrinsic apoptosis was evidenced by elevated caspase-9 and the downstream executioner caspase-3. The activation of these apoptotic mediators provides mechanistic insight into the tubular epithelial cell loss observed histologically (Huang et al., 2022; Ji et al., 2024). This histological pattern is supported by earlier evidence showing that CPA-induced renal injury is closely linked to apoptotic pathway engagement. Buspirone pretreatment markedly reduced caspase-3, caspase-9, and caspase-12 expression, indicating inhibition across both ER stress–related and mitochondrial apoptosis (Alshahrani et al., 2022). The attenuation of apoptotic signaling likely contributed to the observed preservation of renal structure and improvement of functional parameters, consistent with previous reports linking suppression of apoptosis by buspirone to improved tissue integrity and organ function (Khalaf et al., 2026).

Despite these findings, the present study employed a single-dose acute CPA model and therefore primarily reflects the early functional and molecular events of renal injury. Although attenuation of tubular damage, oxidative and ER stress, inflammation, and apoptosis may favor subsequent renal recovery, the current design cannot determine whether the protective effects of buspirone persist after its discontinuation or prevent cumulative renal injury and fibrotic remodeling.

## 5 Conclusion

**FIGURE 12:**
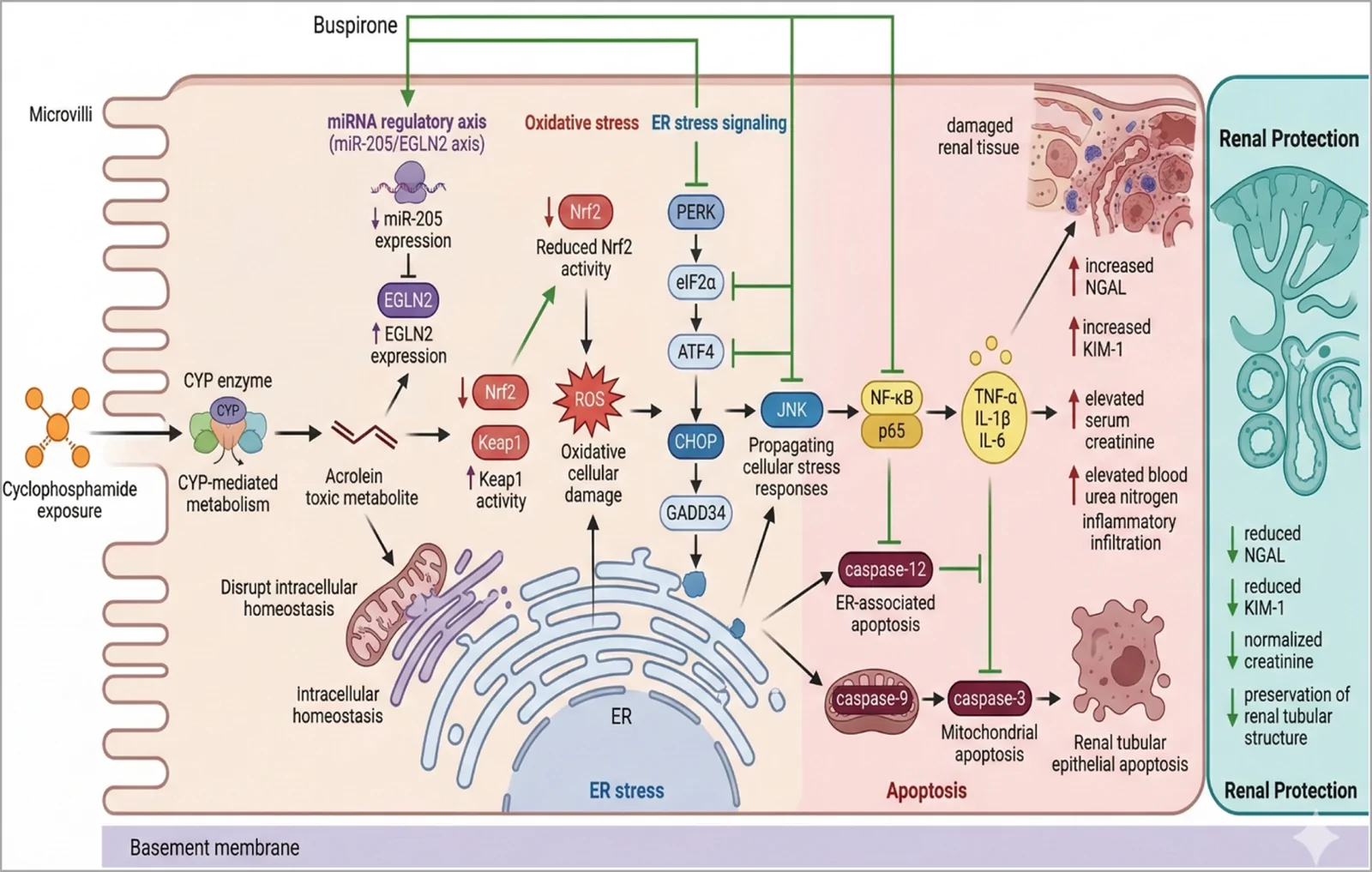
Proposed molecular pathways implicated in buspirone-mediated renoprotection during cyclophosphamide-evoked renal toxicity. Cyclophosphamide induces renal injury via disruption of the miR-205/EGLN2 axis, impairment of Nrf2 signaling, activation of PERK/ATF4/CHOP-dependent ER stress, stimulation of JNK, and induction of NF-κB–driven inflammation, leading to apoptosis. Buspirone administration reverses these effects by restoring miR-205 and suppressing EGLN2, enhancing Nrf2 activity, and attenuating ER stress and inflammatory pathways, thereby limiting apoptosis and preserving renal integrity.

Collectively, the present findings demonstrate that CPA-induced nephrotoxicity is driven by coordinated activation of multiple stress-related pathways, including disruption of the miR-205/EGLN2 axis, impairment of Nrf2 antioxidant defense, activation of PERK/ATF4/CHOP-associated ER stress, stimulation of JNK signaling, induction of NF-κB–mediated inflammation, and engagement of apoptotic cascades. Buspirone effectively mitigated these alterations by restoring miR-205 and suppressing EGLN2, thereby reestablishing cellular homeostasis. By reinforcing Nrf2-dependent antioxidant defense while restraining ER-stress and inflammatory signaling cascades, buspirone reduced apoptotic cell death and preserved renal architecture (Figure 12). These results underscore the preventive renoprotective potential of buspirone against cyclophosphamide-induced renal injury and highlight the relevance of the miR-205/EGLN2 pathway.

## 6 Limitations and future directions

Although the miR-205–EGLN2 interaction has been functionally validated, the absence of direct miR-205 or EGLN2 manipulation in the present model limits the findings to an association with buspirone protection rather than drug-specific causal proof. The lack of a buspirone-only group also prevented assessment of its effects on normal renal function, basal oxidative status, and miR-205/EGLN2 expression, despite its established tolerability and experimental support for the selected doses. Moreover, plasma and renal concentrations of buspirone and its metabolites were not measured, limiting direct clinical dose extrapolation. Future studies should include renal tubular-cell models, miR-205 mimics or inhibitors, EGLN2 manipulation, rescue experiments, buspirone-only groups, and pharmacokinetic–pharmacodynamic analyses. Repeated CPA exposure, recovery-phase assessments, extended follow-up, and clinically relevant models are also needed to evaluate sustained protection, cumulative renal injury, and fibrotic remodeling. Further toxicokinetic and toxicodynamic studies are needed to evaluate buspirone’s safety in tumor treatment settings.

## Data Availability

The original contributions presented in the study are included in the article/Supplementary Material, further inquiries can be directed to the corresponding author.
