# Supplementary material for: Buspirone attenuates cyclophosphamide-induced renal dysfunction in association with alterations in miR-205/EGLN2, Nrf2, and PERK/ATF4/CHOP signaling
Source: Front Pharmacol. 2026 Aug 17;17:1871321. doi: 10.3389/fphar.2026.1871321 (PMC13539122; doi:10.3389/fphar.2026.1871321)
Supplement: Supplementary file 1 [file Supplementaryfile1.docx]

**Title:**

**Buspirone attenuates cyclophosphamide-induced renal dysfunction in association with alterations in miR-205/EGLN2, Nrf2, and PERK/ATF4/CHOP signaling**

**Authors & affiliations:**

Ahmed M. El-Dessouki^1^, Amal Abdullah Alrashidi^2*^, Mohammad Ibrahim Jumaa^3^, Hagar M. El-Sadek^4^, Asmaa Ramadan^5^, Shaimaa fathy mohamed khalil^6^, Sara Youssif Ibrahim^7^, Nada A. Ashour^8^, Samar H. Rizk^9^, Omneya Galal^1^, Nahed A Raslan^10^, Ahmed Mohamed Farghly^10^, Amina Ibrahim Badawy^11^, Shaza M. Elhusseiny^12^, Farha M Shaikh^13, 14^, Wael M Elsaed^15, 16^, Hend A. Abdallah^17^, Hany M. A. Sonpol^18^, Shimaa M Motawei^19^, Eman Hamza^20^

^1^Pharmacology and Toxicology Department, Faculty of Pharmacy, Ahram Canadian University, 12566, Giza, Egypt.

^2^Department of Pharmaceutical Sciences, College of Pharmacy, Princess Nourah bint Abdulrahman University, P.O. Box 84428, Riyadh, 11671, Saudi Arabia.

^3^Department of Anatomy and Physiology, College of Medicine, Imam Mohammad Ibn Saud Islamic University (IMSIU), Riyadh 13317, Saudi Arabia.

^4^Pharmacology and Toxicology Department, Faculty of Pharmacy, October 6 University, Giza 12585, Egypt.

^5^Biochemistry Department, Faculty of Pharmacy, Delta University for Science and Technology, Gamasa 11152, Egypt.

^6^Clinical Pharmacy department, Faculty of Pharmacy, Misr International University (MIU), Cairo 11828, Egypt.

^7^Department of clinical pharmacy and pharmacy practice, Faculty of Pharmacy, Ahram Canadian University, 6^th^ of October City, Giza, Egypt.

^8^Department of Pharmacology and Toxicology, Faculty of Pharmacy, Mansoura National University, Gamasa 7731168, Egypt.

^9^Biochemistry Department, Faculty of Pharmacy, Ahram Canadian University, 6th of October City, Giza, Egypt.

^10^Department of Clinical Pharmacy, College of Health Sciences, Al-Rayan Colleges, AL-Madinah AL-Munawarah City, Madina, P.O 41411, Box 167, Saudi Arabia.

^11^Nursing Department, Al Rayan National College of Health Sciences and Nursing, Al-Madinah Al-Munawarah City, Saudi Arabia.

^12^Microbiology and Immunology Department, Faculty of Pharmacy, Ahram Canadian University, Giza, Egypt.

^13^Department of Basic Medical Sciences, College of Medicine, AlMaarefa University, Diriyah, 13713, Riyadh, Saudi Arabia.

^14^Research Center, Deanship of Scientific Research and Post-Graduate Studies, AlMaarefa University, Diriyah, 13713, Riyadh, Saudi Arabia.

^15^Department of Human Anatomy and Embryology, Faculty of Medicine, Mansoura University, Mansoura, 35516, Egypt

^16^Preclinical Medical Department, College of Medicine & Dentistry, Riyadh Elm University (REU), Riyadh, Kingdom of Saudi Arabia.

^17^Physiology Department, Faculty of Medicine, Benha University, Benha, Egypt.

^18^Department of anatomy and Embryology, Mansoura Faculty of Medicine, Mansoura University, Egypt.

^19^Department of Forensic Medicine and Clinical Toxicology, Faculty of Medicine, Mansoura University, Egypt.

^20^Department of medical biochemistry and molecular biology, Faculty of Medicine, Mansoura University, Mansoura, Egypt.

***Corresponding author:**

Amal Abdullah Alrashidi: E-mail: [aaalrashidi@pnu.edu.sa](mailto:aaalrashidi@pnu.edu.sa)

**Uncropped Western blot images:**

**ATF4**

**CHOP**

**β-actin**

**Western Blot Analysis**

Endoplasmic reticulum stress in renal tissues was evaluated through the determination of activating transcription factor 4 (ATF4) and C/EBP homologous protein (CHOP). Total protein was extracted from cardiac samples by homogenization in ice-cold RIPA buffer supplemented with protease and phosphatase inhibitors (Thermo Fisher Scientific). Homogenates were clarified by centrifugation at 12,000 × g for 20 minutes at 4 °C, and the supernatants were collected for protein analysis. Protein concentration was determined using a bicinchoninic acid (BCA) assay kit (Thermo Fisher Scientific). Equal amounts of protein (30 μg) were separated on 10–12% SDS–polyacrylamide gels and subsequently transferred onto PVDF membranes (Millipore). Membranes were blocked for 1 hour at room temperature with 5% nonfat dry milk or 5% bovine serum albumin in Tris-buffered saline containing 0.1% Tween-20 (TBST). Primary antibodies against activating transcription factor 4 (ATF4; Cat. No. 11815, Cell Signaling Technology) and C/EBP homologous protein (CHOP; Cat. No. 2895, Cell Signaling Technology), both validated for rat samples, were incubated overnight at 4 °C.

Following washing with TBST, membranes were incubated for 1 hour at room temperature with the appropriate horseradish peroxidase (HRP)-conjugated secondary antibodies, including goat anti-rabbit IgG for ATF4 and goat anti-mouse IgG for CHOP (Thermo Fisher Scientific; 1:5,000). Protein bands were visualized using enhanced chemiluminescence (ECL) reagents (Bio-Rad) and captured using a chemiluminescence imaging system. Densitometric analysis was performed using ImageJ software, and protein expression levels were normalized to β-actin (Cat. No. 3700, Cell Signaling Technology), which served as the internal loading control.
